# Low back pain and perceived psychological workload among electricians

**DOI:** 10.1192/j.eurpsy.2024.1351

**Published:** 2024-08-27

**Authors:** I. Sellami, A. Feki, A. Abbes, S. Baklouti, M. L. Masmoudi, K. Jmal Hammami, M. Hajjaji

**Affiliations:** ^1^Occupational medicine, Hedi Chaker Hospital; ^2^ Medicine univeristy; ^3^Rheumatoloy, Hedi Chaker Hospital, Sfax, Tunisia

## Abstract

**Introduction:**

Low back pain (LBP) is a serious threat to electricians. It is well known that LBP is associated with physical strain. But the impact of psychological workload on the occurrence of LBP needs further investigation.

**Objectives:**

This study aimed to assess the link between LBP and perceived psychological workload among electricians.

**Methods:**

The study was conducted with a sample of workers from a Tunisian Electricity society. Data were gathered between January-June 2022 using a self-administered questionnaire including socio-professional characteristics and the Nordic musculoskeletal questionnaire during the last 12 months and the last 7 days. To assess the perceived workload, we used the National Aeronautics and Space Administration Task Load Index (NASA-TLX). In this study, we evaluated raw NASA-TLX scores.

**Results:**

Our study included 68 male electricians. The mean age was 39.2 ± 10.3 years. The average job tenure was 16± 11.4 years. According to the Nordic musculoskeletal questionnaire, 32.4% of participants reported low back pain during the last 12 months. Thirty participants (19.1%) had low back pain during the last 7 days. The mean score of mental demand, physical demand, performance, effort, frustration level and temporal demand were respectively 88.2±14.3, 61.1±24, 84.8±13.3, 82.6±14.5, 35.8±29.2 and 60.4±28.8. The frustration level was associated with the presence of LBP during the last 12 months and the last 7 days (p<0.05).

**Conclusions:**

From the results of this study, we conclude that LBP was associated with the perceived psychological workload. Hence, the prevention of LBP should go through the improvement of work conditions to enhance the mental health of the electricians.

**Disclosure of Interest:**

None Declared

